# Supplementary material for: Spatial variability of microzooplankton grazing on phytoplankton in coastal southern Florida, USA
Source: PeerJ. 2022 Apr 25;10:e13291. doi: 10.7717/peerj.13291 (PMC9048640; doi:10.7717/peerj.13291)
Supplement: Supplemental Information 1 — Comparison of the results from dilution experiments set-up at the three station in the Florida Kays. Recorded data includes, instantaneous phytoplankton growth rate (µ), microzooplankton community grazing coefficient (g), and net accumulation rate (acc). *Refers to µ and g that are statistically different from zero (P < 0.05). [file peerj-10-13291-s001.docx]

| **Station** | **Date** | ***µ* (d^-1^)** | ***g* (d^-1^)** | **acc (d^-1^)** |
| --- | --- | --- | --- | --- |
| MR | 1/8/2018 | 1.03* + 0.05 | 0.93* + 0.14 | 0.10 + 0.15 |
|  | 3/9/2018 | 0.29* + 0.14 | -0.06 + 0.27 | 0.23 + 0.28 |
|  | 4/30/2018 | 0.74* + 0.15 | 0.69 + 0.44 | 0.05 + 0.46 |
|  | 6/22/2018 | 0.55* + 0.20 | 1.81* + 0.59 | -1.26 + 0.62 |
|  | 10/12/2018 | 0.62* + 0.03 | 1.16* + 0.09 | -0.53 + 0.10 |
|  | 12/17/2018 | 0.63* + 0.04 | 0.65* + 0.11 | -0.03 + 0.12 |
|  | 1/28/2019 | 0.54* + 0.08 | 0.81* + 0.22 | -0.27 + 0.23 |
|  | 4/29/2019 | 0.89* + 0.03 | 0.41* + 0.09 | 0.48 + 0.10 |
|  | 7/29/2019 | -0.66* + 0.10 | -0.67 + 0.30 | 0.01 + 0.32 |
|  | 9/23/2019 | 0.52* + 0.13 | 0.01 + 0.39 | 0.51 + 0.41 |
|  | 11/18/2019 | 0.80* + 0.13 | 0.45 + 0.39 | 0.34 + 0.41 |
| LK/21 | 1/9/2018 | 0.95* + 0.07 | 0.59* + 0.22 | 0.36 + 0.23 |
|  | 3/9/2018 | 0.02 + 0.08 | 0.15 + 0.22 | -0.13 + 0.24 |
|  | 5/1/2018 | 0.77* + 0.08 | 1.49 + 0.25 | -0.71 + 0.26 |
|  | 6/23/2018 | 1.03* + 0.09 | 0.48* + 0.27 | 0.55 + 0.29 |
|  | 10/13/2018 | 1.26* + 0.10 | 2.24* + 0.30 | -0.98 + 0.32 |
|  | 12/18/2018 | 0.83* + 0.02 | 0.95* + 0.07 | -0.12 + 0.08 |
|  | 1/29/2019 | 0.38* + 0.04 | 0.19 + 0.11 | 0.19 + 0.12 |
|  | 4/30/2019 | 1.34* + 0.04 | 0.87* + 0.12 | 0.47 + 0.13 |
|  | 7/30/2019 | 0.56* + 0.09 | 0.54 + 0.26 | 0.02 + 0.27 |
|  | 9/24/2019 | 1.20* + 0.24 | 0.90 + 0.72 | 0.30 + 0.76 |
|  | 11/19/2019 | 1.05* + 0.09 | 0.75* + 0.28 | 0.30 + 0.29 |
| WS | 1/9/2018 | 0.69* + 0.03 | 0.57* + 0.09 | 0.12 + 0.09 |
|  | 3/9/2018 | 1.08* + 0.05 | 0.98* + 0.15 | 0.10 + 0.16 |
|  | 5/1/2018 | 0.52* + 0.11 | 0.55 + 0.33 | -0.03 + 0.35 |
|  | 6/23/2018 | 1.14* + 0.06 | 1.03* + 0.18 | 0.11 + 0.19 |
|  | 10/13/2018 | 1.42* + 0.05 | 0.37* + 0.14 | 1.04 + 0.15 |
|  | 12/18/2018 | 0.69* + 0.15 | 0.44 + 0.42 | 0.25 + 0.45 |
|  | 1/29/2019 | 0.70* + 0.02 | 0.35* + 0.07 | 0.35 + 0.07 |
|  | 4/30/2019 | 1.27* + 0.03 | 0.80* + 0.08 | 0.47 + 0.09 |
|  | 7/30/2019 | 0.58* + 0.18 | 0.23 + 0.54 | 0.36 + 0.57 |
|  | 9/24/2019 | 1.65* + 0.13 | 0.86* + 0.37 | 0.78 + 0.39 |
|  | 11/19/2019 | 0.99* + 0.10 | 0.41 + 0.31 | 0.59 + 0.32 |

**SI Table 1. Results from all Florida Keys dilution experiments.**

Comparison of the results from dilution experiments set-up at the three station in the Florida Kays. Recorded data includes, instantaneous phytoplankton growth rate (*µ*), microzooplankton community grazing cofficient (*g*), and net accumulation rate (acc). *Refers to *µ* and *g* that are statistically different from zero (P<0.05).
