# Supplementary material for: Spatial variability of microzooplankton grazing on phytoplankton in coastal southern Florida, USA
Source: PeerJ. 2022 Apr 25;10:e13291. doi: 10.7717/peerj.13291 (PMC9048640; doi:10.7717/peerj.13291)
Supplement: Supplemental Information 2 — Comparison of the results from dilution experiments set-up at the two station in the SWF inner shelf. Recorded data includes, instantaneous phytoplankton growth rate (µ), microzooplankton community grazing cofficient (g), and net accumulation rate (acc). *Refers to µ and g that are statistically different from zero (P < 0.05). [file peerj-10-13291-s002.docx]

| **Station** | **Date** | ***µ* (d^-1^)** | ***g* (d^-1^)** | **acc (d^-1^)** |
| --- | --- | --- | --- | --- |
| 57 | 1/10/2018 | 0.85* + 0.18 | 1.42* + 0.52 | -0.57 + 0.54 |
|  | 3/10/2018 | 0.20* + 0.08 | 0.81* + 0.23 | -0.61 + 0.25 |
|  | 5/2/2018 | 0.01 + 0.48 | -0.39 + 1.26 | 0.40 + 1.35 |
|  | 6/25/2018 | 0.43* + 0.05 | 1.58* + 0.15 | -1.16 + 0.16 |
|  | 8/8/2018 | 0.39* + 0.10 | 0.40 + 0.28 | -0.01 + 0.30 |
|  | 10/18/2018 | 1.41* + 0.03 | 1.47* + 0.07 | -0.06 + 0.08 |
|  | 1/31/2019 | 0.04 + 0.03 | 0.30* + 0.10 | -0.26 + 0.10 |
|  | 5/2/2019 | 0.69* + 0.05 | 0.79* + 0.15 | -0.09 + 0.16 |
|  | 9/28/2019 | 0.72* + 0.05 | 1.02* + 0.14 | -0.30 + 0.15 |
|  | 11/23/2019 | 0.85* + 0.12 | 0.65 + 0.35 | 0.20 + 0.37 |
|  | 1/10/2020 | 1.71* + 0.11 | 1.86* + 0.33 | -0.15 + 0.35 |
| 54 | 1/10/2018 | 0.51* + 0.08 | 0.66* + 0.23 | -0.15 + 0.25 |
|  | 3/10/2018 | 0.87* + 0.12 | 1.60* + 0.36 | -0.73 + 0.38 |
|  | 5/2/2018 | 2.94* + 0.38 | 3.78* + 1.07 | -0.84 + 1.13 |
|  | 6/25/2018 | 0.35* + 0.03 | 0.30* + 0.10 | 0.04 + 0.11 |
|  | 8/8/2018 | 0.73* + 0.03 | 1.20* + 0.09 | -0.47 + 0.10 |
|  | 10/18/2018 | 1.35* + 0.44 | 1.62 + 1.30 | -0.27 + 1.38 |
|  | 1/31/2019 | 0.53* + 0.02 | 0.34* + 0.06 | 0.19 + 0.06 |
|  | 5/2/2019 | 0.40* + 0.09 | 0.94* + 0.27 | -0.55 + 0.28 |
|  | 9/28/2019 | 0.88* + 0.13 | 0.92* + 0.38 | -0.04 + 0.40 |
|  | 11/23/2019 | 1.01* + 0.05 | 0.95* + 0.13 | 0.06 + 0.14 |
|  | 1/10/2020 | 0.71* + 0.09 | 1.04* + 0.27 | -0.33 + 0.29 |

**SI Table 2. Results from all SWF Inner Shelf dilution experiments.**

Comparison of the results from dilution experiments set-up at the two station in the SWF inner shelf. Recorded data includes, instantaneous phytoplankton growth rate (*µ*), microzooplankton community grazing cofficient (*g*), and net accumulation rate (acc). *Refers to *µ* and *g* that are statistically different from zero (P<0.05).
