# Supplementary material for: Spatial variability of microzooplankton grazing on phytoplankton in coastal southern Florida, USA
Source: PeerJ. 2022 Apr 25;10:e13291. doi: 10.7717/peerj.13291 (PMC9048640; doi:10.7717/peerj.13291)
Supplement: Supplemental Information 3 — (A) Standard (expected) dilution experiments results from each station with fitted linear regression line and associated equation (MR—10/12/2018). (B) Zero microzooplankton grazing results with fitted linear regression line and associated equation (MR—3/9/2018). (C) Negative slopes with fitted linear regression line and associated equations (MR—7/31/2019). (D) Dilution experiments with non-linear regression results (MR—11/20/2019). [file peerj-10-13291-s003.pdf]

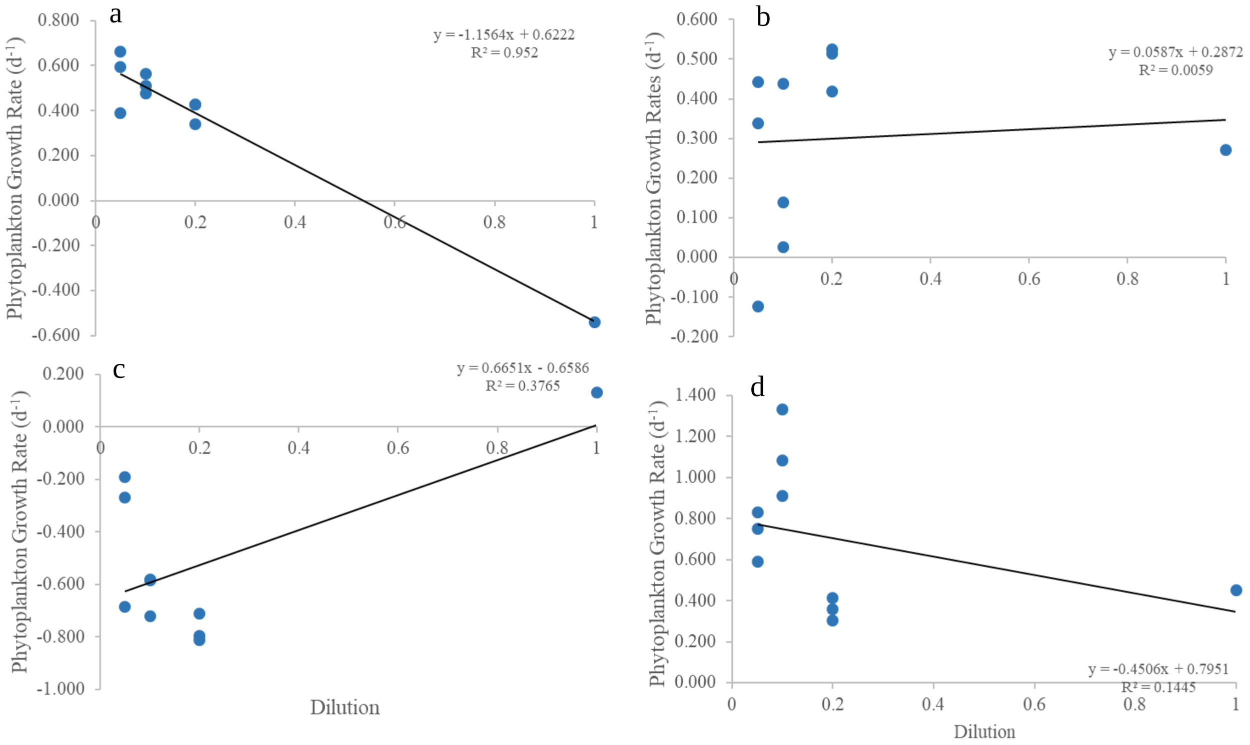

SI Figure 1. Visual representation of different dilution experiment results.

(a) Standard (expected) dilution experiments results from each station with fitted linear regression line and associated equation (MR – 10/12/2018). (b) Zero microzooplankton grazing results with fitted linear regression line and associated equation (MR – 3/9/2018). (c) Negative slopes with fitted linear regression line and associated equations (MR – 7/31/2019). (d) Dilution experiments with non-linear regression results (MR – 11/20/2019).
